# Modeling *Escherichia coli *signal peptidase complex with bound substrate: determinants in the mature peptide influencing signal peptide cleavage

**DOI:** 10.1186/1471-2105-9-S1-S15

**Published:** 2008-02-13

**Authors:** Khar Heng Choo, Joo Chuan Tong, Shoba Ranganathan

**Affiliations:** 1 Institute for Infocomm Research, 21 Heng Mui Keng Terrace, Singapore 119613; 2 Department of Biochemistry, Yong Loo Lin School of Medicine, National University of Singapore, 8 Medical Drive, Singapore 117597; 3 Department of Chemistry and Biomolecular Sciences & Biotechnology Research Institute, Macquarie University, Sydney NSW 2109, Australia

## Abstract

**Background:**

Type I signal peptidases (SPases) are essential membrane-bound serine proteases responsible for the cleavage of signal peptides from proteins that are translocated across biological membranes. The crystal structure of SPase in complex with signal peptide has not been solved and their substrate-binding site and binding specificities remain poorly understood. We report here a structure-based model for *Escherichia coli *DsbA 13–25 in complex with its endogenous type I SPase.

**Results:**

The bound structure of DsbA 13–25 in complex with its endogenous type I SPase reported here reveals the existence of an extended conformation of the precursor protein with a pronounced backbone twist between positions P3 and P1'. Residues 13–25 of DsbA occupy, and thereby define 13 subsites, S7 to S6', within the SPase substrate-binding site. The newly defined subsites, S1' to S6' play critical roles in the substrate specificities of *E. coli *SPase. Our results are in accord with available experimental data.

**Conclusion:**

Collectively, the results of this study provide interesting new insights into the binding conformation of signal peptides and the substrate-binding site of *E. coli *SPase. This is the first report on the modeling of a precursor protein into the entire SPase binding site. Together with the conserved precursor protein binding conformation, the existing and newly identified substrate binding sites readily explain SPase cleavage fidelity, consistent with existing biochemical results and solution structures of inhibitors in complex with *E. coli *SPase. Our data suggests that both signal and mature moiety sequences play important roles and should be considered in the development of predictive tools.

## Background

Translocation across the cell membrane requires the presence of short signal sequences termed "signal peptides" that are localized at the amino terminus (N-terminus) of proteins [1]. These N-termini localized signal peptides are subsequently removed from the newly synthesized precursor proteins by type I signal peptidases (SPases) [2]. SPases play essential roles in the viability of bacteria [3,4], making these enzymes attractive targets for the design of novel antibiotics [5]. Currently, *Escherichia coli *is by far the most widely used host organism for the bacterial expression of heterologous secreted proteins, especially for therapeutic purposes, with reported yields of 5–10 g/L [6]. Mutations in the signal peptide have been known to affect secretion either by enhancing the processing of the cleavage site or by inhibiting this proteolytic processing [7]. It is well known that besides the signal peptide, the N-terminal region of the mature protein also affects the protein secretion [8]. We are therefore interested in understanding the determinants involved in signal peptide recognition, binding and cleavage.

The *E. coli *type I SPase is of particular interest in the study of type I SPases, as its active site is relatively accessible at the bacterial membrane surface [5,9-11]. Although many mutational and biochemical studies have been performed, basic questions such as SPase fidelity and substrate specificity remain unanswered. Signal peptides exhibit limited primary sequence homology, but are well conserved at residues positioned -3 (P3) to -1 (P1) relative to the cleavage site, designated P1-P1' [12]. Comparative analysis of 36 prokaryotic signal peptides reveals that type I SPases specifically recognizes substrates with small neutral residues at both the P3 and P1 positions [13]. P3 is dominated by the presence of alanine, glycine, serine, threonine and valine; while P1 is characterized by alanine, glycine, serine and threonine [12,13]. Accordingly, the P3 and P1 positions have been proposed to constitute the SPase cleavage site and have been actively applied by various groups for predicting signal peptide cleavage sites [12,14]. These findings were cited as affirmation of the location of two key determinants within the signal peptide cleavage site. Unfortunately, no solution structures exist that can illustrate precisely how the precursor protein is oriented within the SPase substrate-binding site prior to proteolysis, or the identity of other critical determinants that control substrate specificity [15].

In this paper, we report the modeling of an *E. coli *periplasmic dithiol oxidase, DsbA 13–25 in complex with *E. coli *type I SPase based on the crystal structures of *E. coli *SPase in complex with β-lactam [16] and lipopeptide [17] inhibitors. The DsbA 13–25 precursor protein was selected for this study due to its efficient periplasmic secretion [15]. By threading the P7 to P1' positions against the solved structures of β-lactam [16] and lipopeptide [17] inhibitors, our model reveals that precursor protein is bound to *E. coli *type I SPase with a pronounced twist between positions P3 and P1'. Thirteen subsites S7 to S6' were identified that might be critical to these and other aspects of catalysis. Our model was additionally corroborated by comparative analysis of 107 experimentally validated substrates.

## Results and discussion

### Substrate binding site

**Figure 1:**
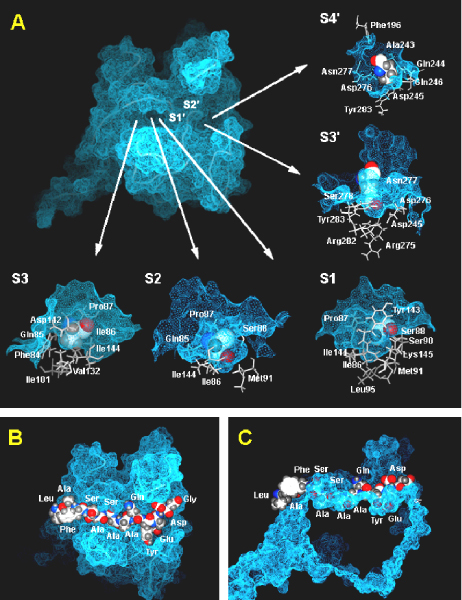
**The *E. coli *type I signal peptidase substrate-binding site**. Pockets defining the binding site of *E. coli *type I signal peptidase. A) Top view of the molecular surface of *E. coli *SPase binding site (colored blue) with Cα trace of SPase (blue lines). Pockets that accommodate signal peptide side chains are shown in detail in surrounding views and numbered in accordance to their position along the peptide from the S1 pocket that contains the active-site nucleophile, Ser90. B) Top view of the molecular surface of *E. coli *SPase binding site (colored blue) with the bound conformation of DsbA precursor peptide as a CPK model. C) Side view of structure in *B*, rotated by 90°.

The energetically favoured and most frequently populated bound conformation of DsbA 13–25 H_2_N-LAFSASAΔAQYEDG-COOH, where the cleavage site is indicated by Δ [18], to *E. coli *type I SPase was obtained from our structural model. The complex defines 13 enzyme subsites, S7 to S6', within the SPase substrate binding site, interacting with bound precursor SPases. Among these, six smaller clefts or 'pockets' were identified at subsites S3, S2, S1, S1', S3' and S4' respectively (Figure 1). The narrow clefts at S3, S2, S1 and S1' play direct roles in the high specificity of the signal peptide residues while the larger clefts at S3' and S4' may be responsible for the specificity of the mature moieties.

**Figure 2:**
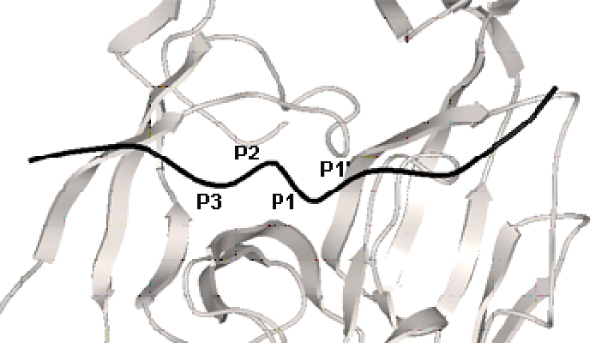
**A model of the DsbA 13–25 in complex with *E. coli *SPase**. A model of the DsbA 13–25 precursor protein (Cα trace in black) bound to the active site of *E. coli *SPase (schematic cartoon diagram in grey) illustrating a pronounced twist in the peptide backbone between P3 and P1' at the catalytic site.

The side chain of Ala19 (P1, Figure 1A) is buried within the S1 subsite, which is composed of primarily hydrophobic and non-polar enzyme residues including the previously identified Ile86, Pro87, Ser88, Ser90, Met91, Leu95, Tyr143, Ile144 and Lys145 [9,19]. The S2 subsite (Gln85, Ile86, Pro87, Ser88, Met91 and Ile144) constitutes the deepest cavity within the substrate-binding site. This pocket can accommodate residues with large side chains and appears to play an important role in substrate specificity of *E. coli *type I SPase, consistent with biochemical experiments (discussed in *Substrate specificity*). This subsite, formerly proposed as the S1 subsite by Paetzel *et al*. [9,19], largely overlaps with the S1 subsite due to a pronounced twist in the P3 to P1' binding conformation (Figure 2; detailed in *Substrate binding conformation*). Our model reveals that the Ser18 (P2) side chain is not solvent exposed but is completely buried at this location, due to a pronounced twist in the P3 to P1' binding conformation (Figure 2; detailed in *Substrate binding conformation*). The S3 subsite, which is composed of non-polar atoms from residues Phe84, Gln85, Ile86, Pro87, Ile101, Val132, Asp142 and Ile144 [9,19], is located diagonally across from the S1 subsite. This pocket constitutes the third deepest cavity within the substrate-binding site and can accommodate a wide variety of side chains. The S4 subsite consisting of Phe84, Gln85, Pro87 and Asp142, is in contact with Ser16 (P4). Further upstream, the S5 subsite is defined by Phe84, Gln85 and Asp142; S6 consists of Pro83 and Phe84; while the S7 subsite includes Glu82 and Pro83.

**Figure 3:**
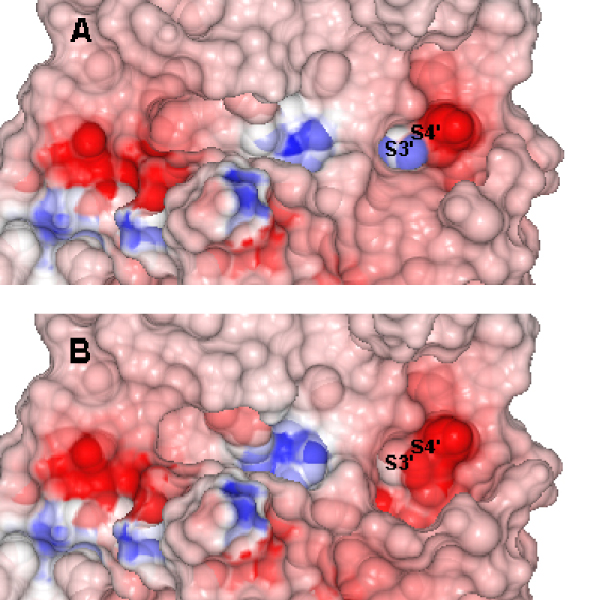
**The S3'/S4' subsites of *E. coli *SPase**. Rearrangements of side chain residues at S3'/S4' subsites in the crystallographic structure of *E. coli *type 1 SPase (PDB ID: 1B12). *A*, The side chain of Asp276 is exposed to interact with amino acid residues at positions -3 and -4. *B*, Rearrangements of Asp276 and Arg282 result in a positively charged pocket at S3'/S4' subsites.

At the P1' to P6' positions of the mature moiety, our model indicates that the side chains of P1' to P5' residues are in position to make significant contact with the *E. coli *type I SPase. The S1' subsite shares similar residues with the S1 subsite and includes Ser88, Ser90, Tyr143 and Ala279. The S2' subsite includes Ser88, Ser90, Phe208, Asn277 and Ala279. The S3' and S4' subsites constitute a broad pocket that can accommodate both positive and negative charged residues by rearrangements of side chains (Figure 3). The S3' subsite is composed of Met249, Tyr50, Asp276, Asn277, Ala279, Arg282 and Tyr283, while the S4' subsite includes Gln244, Asp245, Asp276, Asn277 and Arg282. Further downstream, the S5' subsite consists of Phe196, Ser206, Ala243, Asp276 and Asn277, while the S6' subsite includes Phe196, Ile242 and Ala243.

In our model, the bound precursor protein makes significant contact with *E. coli *SPase I from S7 to S6'. Models described earlier only focused on the P3-P1' segment and did not analyze in full the different substrate binding pockets on either side of the scissile bond. In particular, the S2 subsite was formerly proposed as the S1 subsite by Paetzel *et al*. [9,19] as it largely overlaps with the latter. In contrast to the analysis by Paetzel *et al*. [19], our model reveals that the Ser18 (P2) side chain is not solvent exposed but is completely buried at this location. The ability of S3'/S4' to alter their electrostatic requirements by varying side chain conformations (Figure 3) may help explain the propensity to find substrates with charged amino acids at these positions (discussed in detail in *Substrate Specificity*).

### Substrate binding conformation

**Figure 4:**
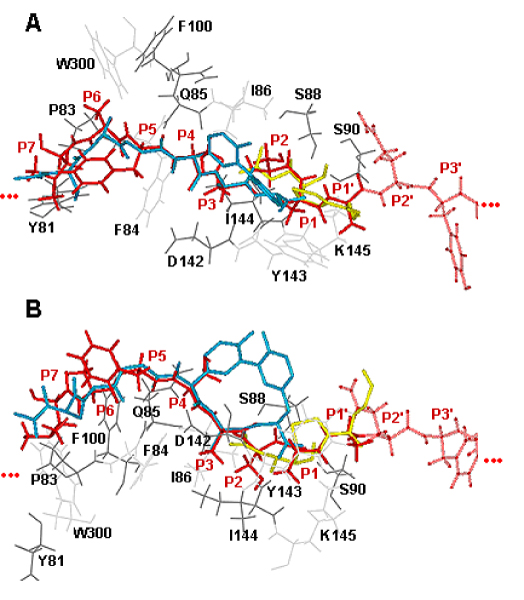
**Superimposition of DsbA 13–25 precursor protein with lipopeptide and β-lactam inhibitors**. A model of the DsbA 13–25 precursor protein (red) bound to the active site of *E. coli *type 1 SPase (grey). Superimposition of the P7 to P1' positions of DsbA precursor protein with the lipopeptide (blue; PDB ID: 1T7D) and β-lactam (yellow; PDB ID: 1B12) inhibitors from *A*, top view and *B*, side view respectively. Residues N-terminal to P7 and C-terminal to P2' have been truncated for clarity.

We have modeled the coordinates of P7 to P1' by threading against the solved structures of β-lactam [16] and lipopeptide [17] inhibitors in complex with *E. coli *SPase, followed by *ab initio *docking of the P2' to P6' positions (for more details, refer *Methods*). The precursor protein, DsbA 13–25 is bound to *E. coli *type I SPase in an extended conformation with a pronounced backbone twist between Ala17 (P3) and Ala20 (P1') (Figure 2). In the P3-P1' segment, the first three side chains are all oriented towards the binding groove, and only the P1' side chain oriented across the binding groove. As shown in Figure 4, similar interactions between the *E. coli *type 1 SPase with DsbA 13–25 model, lipopeptide inhibitor (PDB ID: 1T7D) and β-lactam inhibitor (PDB ID: 1B12) were observed. The conformation of P3' and P4' allow their corresponding side-chains to extend into a large cavity (S3'/S4' subsite; Figure 1). As such, medium or large residues are preferred at these two positions for favorable interactions. Good agreement with the known experimental data (refer Substrate Specificity) is obtained, supporting the validity of our model.

Ten positions for hydrogen bonding were identified supporting high affinity binding between *E. coli *SPase and DsbA 13–25. These include Ser18 (P2) O...Ser88 NH, Ser18 (P2) O...Ser88 OG, Ala19 (P1) N...Ser88 OG, Gly89 N...Gln21 (P2') OE1, Ala19 (P1) N...Ser90 OG, Ser90 OG...Ala20 (P1') O, Lys145 NZ...Ala19 (P1) O, Gln194 NE2...Asp24 (P5') OD2, Ser206 OG...Asp24 (P5') OD2 and Arg282 NH1...Glu23 (P4') OE1. Our model suggests that the enzyme-substrate contact points extend all the way from P7 to P6' of the DsbA precursor protein.

The orientation of DsbA 13–25 side chains within the active site (P7-P6') of *E. coli *SPase adopts the pattern [20]: ↓ • • • ↓ ↓ ↓ • • ↓ ↓ • • (where ↓ represents a side chain oriented towards the binding site and • represents a side chain oriented away or across the binding site). Specifically, the P3-P1' segment adopts the pattern: ↓ ↓ ↓ •, with the side chains of P3, P2 and P1 oriented towards the binding groove thereby supporting the stringent selectivity criteria in this region. The side chain of P1' alone is oriented differently, in accord with the observed variability in this position. A similar conformation was obtained for the precursor sequence OmpA 15–27 [21,22] H_2_N-FATVAQAΔATSTKK-COOH (P1-P1' cleavage site indicated by Δ) in complex with *E. coli *type 1 SPase (data not shown). Here again, the P3-P1' side chains of OmpA adopt the orientation ↓ ↓ ↓ •, while the model proposed by Paetzel *et al*. [19] and Ekici *et al*. [22], adopts the pattern ↓ • ↓ •, with the side chain of P2 not pointing towards the binding groove. The disparity between our model and Paetzel *et al*. [19] may be attributed to the selection of different template structures where the structures of the covalently bound peptide inhibitor complex and the analogous enzyme LexA were used to guide the P1 and P3 to P6 positions of the later [19], while the coordinates of P7 to P1' for our model were guided by the solved structures of β-lactam [16] and lipopeptide [17] inhibitors in complex with *E. coli *SPase. In our models, the P2 side chains in the bound DsbA and OmpA models are hydrogen-bonded to the catalytically important SPase I residue, Ser88 [23]. The twist in the backbone conformation in the region P3-P1' is representative of the transition state, with three critical hydrogen bonds conserved in our model and the bound β-lactamase and lipoprotein inhibitors, with the atoms Ser88 Oγ, Ser90 Oγ and Lys145 Nζ important for catalytic activity.

### Substrate specificity

**Figure 5:**
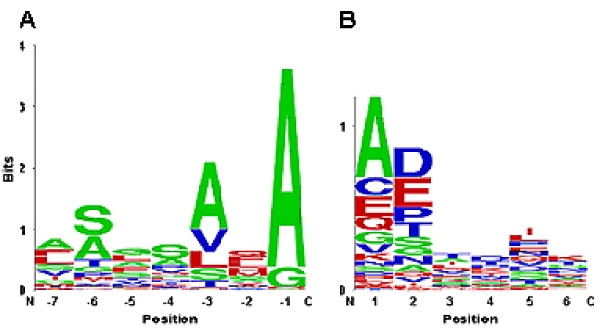
**Analysis of *E. coli signal sequences***. Sequence logo [35] illustrating the size (small: green; medium: blue; large: red) of amino acids at different positions along the precursor proteins of 107 experimentally verified *E. coli *signal sequences from SPdb, showing *A*, the end of the signal peptide (P7-P1) and *B*, the start of the mature moiety (P1'-P6').

We were interested in understanding how the peptides modeled in this study reflect the *E. coli *repertoire of secreted signals. Comparative analysis of 107 experimentally determined *E. coli *type I SPase substrates (Figure 5) revealed high conservation of amino acid residues at positions P3 and P1. In particular, position P1 is dominated by small (99%), hydrophobic (98%), and neutral (100%) residues, with alanine being the predominant residue (92% or 98/107) at this position, followed by glycine (9%). Position P2 shows a strong preference for bigger side chains with 87% (93/107) possessed by medium- or large-sized residues at this location. Position P3 also shows a preference for hydrophobic residues (83%). Although this position contains mainly small amino acid residues (61%), it can also accommodate both medium (25%) and large (14%) residues. Only 50% (54/107) of the sequences contain the consensus Ala-X-Ala recognition motif, while even fewer sequences (18%; 19/107) contain a Val-X-Ala recognition sequence. In our modeled structure for DsbA propeptide, the side chains from P7 to P4 are also in positions to make substantial contacts with SPase (Figure 2), but are not confined to 'pockets'. Nonetheless, these residues may also participate in binding by interacting with surface residues of SPase. These observations are in accord with the lack of residue preference observed in these positions (Figure 5). Overall, in the 107 signal peptide dataset, neutral residues (≥98%) are preferred in positions P7 to P1, indicating that charged interactions between signal peptides and *E. coli *type I SPase are disfavored in this stretch, consistent with earlier reports on the carboxy-terminus of the C-region [24]. However, few signal sequences possess polar residues at the C-region (P7: 17%; P6: 48%; P5: 30%; P4: 52%; P3: 18%; P2: 41%; P1: 2%), in contrast with earlier studies [24,25]. Most residues are tolerated at the +1 (P1') position of the mature moiety, with the exception of the large hydrophobes, Ile, Met, Trp, Pro and Arg. Here, Pro is disfavored as the rigid positioning of its backbone hinders docking interactions with SPase at positions P2' to P6'. The majority of *E. coli *signal peptides contain medium or large residues both at P3' (81%) and at P4' (90%) positions. The propensity for negatively charged residues to occur at P3' and P4' positions are low, with observed values of 10% (or 11/107) and 19% (or 20/107) respectively, while 8% (9/107) and 13% (14/107) respectively of the sequences analyzed have positively charged residues at these positions.

## Conclusion

We have developed a structural model for *E. coli *SPase I complex, with bound DsbA and OmpA propeptides, consistent with existing biochemical data and solution structures of *E. coli *type I SPase-inhibitor complexes [16,17]. The developed models provided an opportunity to examine the bound structure of *E. coli *type I SPase complex that have been difficult to solve experimentally. It appears that both signal and mature moiety sequences play direct role in catalysis. This work advances our understanding of the molecular mechanism governing signal peptide specificities and SPase fidelity, it also serves as a useful guide for designing suitable signal and mature peptide sequences for enhancing heterologous protein expression using *E. coli *as the host organism.

## Methods

### Precursor protein sequence data

The SPdb database [26] was used as a source for the *E. coli *type I SPase substrates. A set of 107 precursor protein sequences was extracted and used in the current analysis where sequences with 80% sequence identity were extracted using the CD-HIT software [27] to reduce redundancy and bias [28].

### Crystallographic data

The atomic coordinates of *E. coli *type I SPase were extracted from the Protein Databank (PDB) entry 1B12 [16] which has a 1.95 Å resolution structure. Atomic coordinates for *E. coli *type I SPase-bound β-lactam [16] and lipopeptide [17] inhibitors were retrieved from PDB entries 1B12 and 1T7D respectively. The structures were relaxed by conjugate gradient minimization, using the Internal Coordinate Mechanics (ICM) software package [29].

### Substrate modeling

The coordinates for the P7 to P1' positions of DsbA 13–25 were obtained by threading against the crystallographic structures of *E. coli *type I SPase-bound inhibitors [16,17]. The P7 to P3 positions were taken from the structure of *E. coli *type I SPase-bound lipopeptide inhibitor [17] by substituting the atoms N1, C2, C5, O6, N7, C8, C10, O11, N12, C13, C14, O15, N16, C18, C26, O27, N28, C29, C31, O32 and N33 with DsbA 13–17 main-chain atoms; while coordinates for the P2 to P1' positions were guided by the solution structure of the *E. coli *type I SPase-bound β-lactam [16] inhibitor based on the atoms N4, C5, C6, C3, C9, O10, C15, C16, O17, C18, O19, C20. A flexible docking using biased Monte-Carlo procedure [29-31] that incorporates the Rapid Exact-Boundary ELectrostatics (REBEL) algorithm for evaluation of the electrostatic solvation energy [32] was subsequently performed to sample different conformations and orientations of P2' to P6' positions with respect to the receptor. In each iteration, a random move in the P2' to P6' positions of the ligand was performed and new conformations were selected based on the Metropolis criterion with a temperature of 5000 K [31,33]. The simulation was terminated after 20,000 energy evaluations [31] and the results analyzed for consistency.

### Intermolecular hydrogen bonds

The number of intermolecular hydrogen bonds was calculated using HBPLUS [34] in which hydrogen bonds are defined in accordance with the standard geometric parameters.

## Competing interests

The authors declare that they have no competing interests.

## Authors' contributions

KHC and JCT carried out the computational simulation studies and drafted the manuscript. KHC, JCT and SR participated in the design of the study and interpretation of data. SR developed the project and finalized the manuscript.
